# Matrix-Dependent Selection of Non-Thermal Preservation Strategies for Edible Mushrooms and Highly Perishable Produce: Quality Targets, Mechanistic Evidence, and Scale-Up Readiness

**DOI:** 10.3390/foods15213989

**Published:** 2026-11-06

**Authors:** Jinshuang Yang, Tingjia Deng, Lin Min, Peiyuan Chen, Xing Liu, Qianwang Zheng, Yuan Zou, Hongbo Fan, Bai-Xiong Chen

**Affiliations:** 1 School of Bioengineering, Zunyi Medical University, Jinwan Road No. 368, Zhuhai 519090, Chinaliuxingas@126.com (X.L.); 2 Institute of Food Biotechnology, College of Food Science, South China Agricultural University, Guangzhou 510642, China; zhqianw@scau.edu.cn (Q.Z.); zouyuan@scau.edu.cn (Y.Z.); 3 Xinjiang Fruit Deep-Processing, Storage, and Transportation Engineering Technology Research Center, College of Food Science and Pharmacy, Xinjiang Agricultural University, Urumqi 830052, China

**Keywords:** non-thermal processing, edible mushrooms, fresh-cut produce, matrix effects, hurdle technology, food safety, scale-up, omics

## Abstract

Non-thermal preservation outcomes depend on food structure as well as treatment intensity. This review examines edible mushrooms as the principal case and uses berries and fresh-cut produce as comparator matrices. Power ultrasound, atmospheric cold plasma, plasma-activated water, and acidic electrolyzed water are assessed against microbial control, browning, texture, and water retention; high-pressure processing and pulsed electric fields are considered mainly to define applicability boundaries. Primary food studies are distinguished from isolated-enzyme experiments, liquid matrix studies, and mechanistic hypotheses. In mushrooms, washing treatments can improve selected storage quality endpoints, but enzyme inactivation in buffer does not establish preservation of intact tissue. In berries, skin integrity and softening constrain acceptable exposure; in fresh-cut produce, exposed surfaces facilitate treatment but release organic matter that consumes active species. Combined treatments may provide complementary effects, whereas synergy requires comparison with an additive expectation. Omics measurements identify treatment-associated responses but do not establish a conserved Ca^2+^–MAPK–transcription factor pathway across plants and fungi. A matrix–target–readiness framework links treatment selection to the measured endpoint, quality penalty, and validation context. Translation requires matched safety–quality studies, viability-informed microbial assessment, treatment uniformity measurements, repeated-batch testing, resource accounting, and jurisdiction-specific regulatory review.

## 1. Introduction

Postharvest deterioration reduces food quality and contributes to losses during storage and distribution [1,2]. Thermal treatments control microorganisms and enzymes but can alter texture, flavor, and heat-sensitive constituents [3,4,5,6,7], whereas chemical preservatives carry use restrictions and consumer safety considerations, as illustrated by sulfite sensitivity [8,9].

Edible mushrooms, including *Agaricus bisporus*, *Lentinula edodes*, and *Pleurotus ostreatus*, are low-energy foods that supply dietary fiber, nutrients, and bioactive compounds [10]. Ergothioneine, polysaccharides, and triterpenoids have attracted interest for their biological activities, although findings from experimental systems do not establish health benefits from consuming preserved foods [11,12,13]. The exposed hyphal structure, high moisture content, and continuing metabolism of fresh mushrooms make them susceptible to water loss, softening, and microbial growth [14], whereas the cuticular barriers of berries and the wounded surfaces of fresh-cut produce impose different constraints on treatment access and tissue damage [15].

This review examines how food structure and preservation targets influence the selection of non-thermal treatments for edible mushrooms, with berries and fresh-cut produce used as comparators. It links matrix-specific deterioration to treatment mechanisms, combined washing systems, host responses, and engineering requirements, and it treats results from juices, purées, and purified enzymes as mechanistic evidence rather than a substitute for experiments on the intended food. Table 1 collates the primary food studies discussed below.

This is a narrative review of primary food studies and the relevant mechanistic literature. A targeted Europe PMC search on 17 October 2026 combined terms for mushrooms, berries, and fresh-cut produce with the technologies and preservation endpoints discussed here; studies were selected according to the food matrix, treatment conditions, measured endpoint, and comparator. Food experiments, isolated-enzyme and liquid matrix studies, and process-scale validation were assessed separately. This review was not designed as a systematic review or meta-analysis.

## 2. Matrix-Specific Deterioration Pathways and Preservation Targets

The three commodity groups share several deterioration endpoints but differ in tissue architecture. Mushrooms have porous chitin–glucan tissues, berries retain species-dependent epidermal and cuticular barriers, and fresh-cut produce has exposed, wounded surfaces. These differences influence enzyme–substrate contact, water loss, tissue softening, and microbial access.

### 2.1. Enzymatic Browning: Molecular Basis and Disruption of Subcellular Compartmentation

Enzymatic browning affects the appearance of mushrooms and fresh-cut plant tissues and involves phenolic oxidation by polyphenol oxidase (PPO) and peroxidase (POD) [16,17]. In plants, cutting can disrupt the separation between phenolic substrates and oxidative enzymes; PPO localization in plastids has been documented in apple tissue [18]. Fungal cells lack plastids, so the relevant cellular organization differs in mushrooms. Injury can nevertheless alter enzyme–substrate contact, oxygen access, and redox conditions in both groups.

PPO belongs to the type-3 copper protein family, whose binuclear copper center supports phenol oxidation. Substrate specificity, residue numbering, and latent forms vary among proteins and organisms [19,20,21,22,23]. Three catalytic states are commonly described: deoxy, oxy, and met.

(1) Deoxy-PPO ([Cu(I)–Cu(I)]): possesses strong oxygen-binding activity.
(2) Oxy-PPO ([Cu(II)–O2^2−^–Cu(II)]): the active form bound to a peroxide ion, primarily catalyzing the ortho-hydroxylation of monophenols (cresolase activity) [24,25], producing o-diphenols.
(3) Met-PPO ([Cu(II)–Cu(II)]): cannot directly bind oxygen; it mainly catalyzes the dehydrogenation oxidation of o-diphenols into highly chemically reactive o-benzoquinones via diphenolase activity (catecholase activity) [23,26].

Some PPOs occur in latent forms that can be activated by structural changes or proteolysis. Studies of plant PPOs have characterized the contribution of the C-terminal domain to enzyme accessibility [19,27]. Activation within postharvest tissue also depends on the local enzyme form and cellular environment.

Protease-dependent activation of latent *Agaricus bisporus* tyrosinase has been demonstrated in vitro [28], supporting a possible role for proteolysis in fungal PPO activation. The importance of this route during storage depends on the tissue conditions and enzyme forms present. Gene family analyses provide candidate PPOs for investigating these relationships in mushrooms [29].

PPO-generated quinones can undergo non-enzymatic reactions with other phenolics and nucleophilic groups in proteins, contributing to brown pigments [16,30]. Preventing visible browning therefore requires attention to substrate availability and tissue integrity as well as residual enzyme activity.

In fresh-cut plant tissues, phenylalanine ammonia-lyase (PAL) can increase phenolic supply after wounding, while POD uses hydrogen peroxide to oxidize phenolic substrates [31,32]. The balance between phenolic synthesis, oxidation, and tissue leakage therefore influences visible browning. Reducing PPO activity alone may not preserve appearance if the treatment also increases substrate release or membrane damage. Plant phenylpropanoid responses require separate consideration from fungal browning mechanisms.

### 2.2. Commodity-Specific Microbial Hazards, Biofilms, and Mycotoxin Risk

Edible mushrooms lack a protective cuticle, whereas cutting or mechanical damage disrupts the epidermis of fresh produce and berries. Exuded juices then create a surface microenvironment rich in reducing sugars, free amino acids, and inorganic salts that favors microbial colonization and proliferation [33].

Mushroom-associated microorganisms include spoilage organisms and foodborne pathogens [33]. *Pseudomonas tolaasii*-associated browning and *Listeria monocytogenes* growth or persistence in mushroom products represent different quality and safety concerns [34,35]. Extracellular polymeric substances (EPS) promote attachment and biofilm-associated persistence on produce [36]. Biofilm protection varies with the organism, surface, and treatment, making food surface studies more informative for washing performance than planktonic cell assays alone.

Mycotoxin occurrence depends on the producing organism, commodity, product form, and storage conditions [37]. Spoilage alone does not establish a mycotoxin hazard. In fresh mushrooms, risk assessment requires evidence of the relevant toxigenic organism and toxin rather than extrapolation from contaminated fruit, vegetables, or dried products.

Microbial inactivation and toxin degradation are distinct preservation endpoints. A decline in the parent toxin concentration does not establish detoxification unless transformation products and residual toxicity are also evaluated.

### 2.3. Cell Wall Depolymerization, Transpiration, and the Lipid Peroxidation Cascade

Postharvest water loss reduces turgor and promotes shriveling. The loss that can be tolerated before marketability declines depends on the commodity, storage temperature, relative humidity, and quality criterion; a single percentage threshold is therefore unsuitable across products.

Oxidative stress can accompany postharvest deterioration. Reactive oxygen species (ROS) include both radicals, such as hydroxyl and superoxide, and non-radical species, such as hydrogen peroxide and singlet oxygen [38,39]. Lipoxygenase-associated oxidation of unsaturated lipids and subsequent breakdown products can be linked with membrane damage in plant tissues [40]. Electrolyte leakage is a useful tissue integrity indicator, including in mushroom storage studies, but it is not a direct measure of microbial safety or a specific signaling pathway [41].

Edible mushrooms and plant-derived produce differ substantially in cell wall composition and in the mechanisms that drive softening [42]. In berries and fresh-cut plant tissues, the cell wall consists mainly of pectin, cellulose, and hemicellulose. During postharvest ripening and senescence, polygalacturonase (PG, EC 3.2.1.15) and pectin methylesterase (PME, EC 3.1.1.11) contribute to pectin modification, while endo-β-1,4-glucanase (Cx) acts on cell-wall glucans. These cell-wall changes can contributes to softening and loss of crispness.

Mushroom cell walls consist principally of chitin and glucans, whereas pectin degradation contributes to softening in plant tissues. Mushroom preservation studies have linked changes in browning and microbial load with biochemical indicators of tissue condition [43,44]. Firmness, weight loss, and membrane integrity provide complementary measures of fungal tissue deterioration, whose enzymatic basis differs from that of plant cell walls.

**Figure 1:**
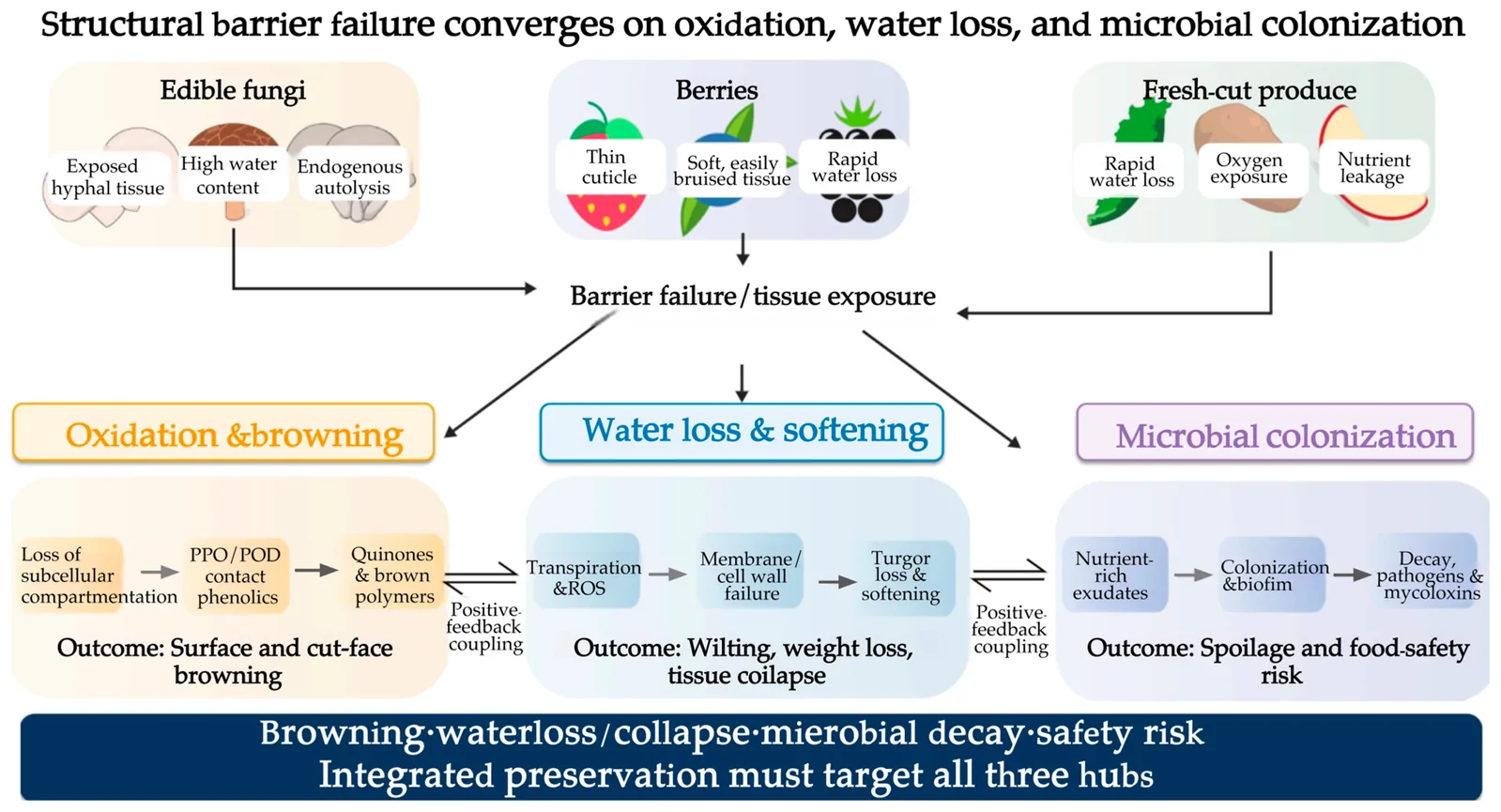
Structural vulnerabilities and coupled deterioration pathways in edible mushrooms, berries and fresh-cut produce. Barrier failure or tissue exposure links oxidation and browning, water loss and softening, and microbial colonization; double-headed arrows indicate potential feedback. Pathogen and mycotoxin hazards depend on the organism, commodity and storage conditions. PPO/POD, polyphenol oxidase/peroxidase; ROS, reactive oxygen species.

The matrix-specific vulnerabilities converge on oxidation and browning, water loss and softening, and microbial colonization (Figure 1). Feedback among these processes links quality deterioration with safety concerns. Their relative importance varies by commodity, and pathogen or mycotoxin hazards depend on the organisms and storage conditions involved.

## 3. Technology–Matrix Fit of Non-Thermal Preservation Approaches

Non-thermal technologies use distinct physical or chemical agents to alter enzymes, microorganisms, membranes, or mass transfer at low bulk temperatures. Their usefulness is matrix- and endpoint-specific: a treatment effective in juice, purée, or a cut surface cannot be assumed to perform similarly in an intact porous mushroom or whole berry. The evidence discussed below differs in kind, and each subsection identifies food-level storage studies, isolated-enzyme or liquid matrix experiments, and proposed mechanisms separately.

### 3.1. Power Ultrasound: Cavitation-Mediated Enzyme Inactivation and Ice Crystal Control

Power ultrasound (US) generates acoustic cavitation, producing localized mechanical and chemical effects [45]. Shear, microjets, and acoustic streaming affect cells, surfaces, and mass transfer, while radical formation adds a chemical component [46,47]. Conditions near collapsing bubbles differ from the bulk temperature and pressure of the food. Treatment performance consequently depends on frequency, delivered power, temperature, exposure time, and equipment geometry.

Ultrasound affects both enzyme structure and activity. Xu et al. treated commercial mushroom PPO in phosphate buffer using flat sweep frequency and pulsed ultrasound [47]. At 50 °C for 30 min, the 22/40 kHz treatment left 1.47% residual activity, and structural changes accompanied the activity loss, consistent with thermosonication-induced protein alteration. Because the experiment used an enzyme preparation, food-tissue and microbial measurements are needed to establish preservation relevance. The relationship between protein conformation and inactivation also varies with enzyme and treatment conditions [48].

Ultrasound-assisted freezing has a different objective from washing followed by refrigerated storage. CO_2_ pressurization combined with ultrasound has been investigated for the freezing quality of fresh-cut honeydew melon [49]. In this application, nucleation, cooling history, ice structure, and post-thaw quality are the relevant endpoints. Their response depends on the commodity and cannot be predicted from refrigerated washing results.

### 3.2. Cold Plasma and Plasma-Activated Water: Reactive-Species Mechanisms

Atmospheric cold plasma (ACP) comprises non-equilibrium gas discharges operated near atmospheric pressure, including plasma-jet configurations [50]. Direct treatment exposes food in or near the discharge; indirect approaches use post-discharge gases or plasma-activated liquids. Plasma-activated water (PAW) is water exposed to a discharge before or during treatment. Differences in contact geometry and chemistry require mode-specific dose characterization. Reactive oxygen and nitrogen species (RONS) include radicals and non-radical oxidants; singlet oxygen and ozone are non-radical species. Cold plasma research on aflatoxin control shows that oxidative degradation is possible [51], but pathways and product safety remain toxin- and treatment-specific.

PAW chemistry varies with discharge conditions, gas and water composition, storage, and the interval between activation and food contact. Relatively persistent hydrogen peroxide, nitrite, and nitrate can participate in reactions that generate transient species. Peroxynitrite-related chemistry has been implicated in particular PAW systems [52]. The contribution of these transient oxidants depends on their formation and decay near the treatment target.

PAW treatment has affected microbial counts and storage quality in button mushrooms [53,54]. Its performance depends on both water activation and subsequent food contact; longer activation does not necessarily imply longer washing. Cold-plasma pretreatment before hot-air drying of shiitake mushrooms addresses a different process objective [55], for which drying behavior and dried-product quality take precedence over fresh-market characteristics.

PAW composition and storability influence the dose delivered to food [56]. Residue assessment requires measured nitrite and nitrate concentrations and the applicable product-specific limits. Excessive oxidant exposure may also impair sensory or nutritional quality [57]. Establishing a useful treatment window therefore requires microbial, tissue quality, and chemical residue measurements under the intended process conditions.

### 3.3. Slightly Acidic Electrolyzed Water: Antimicrobial Washing and Organic Load Constraints

The antimicrobial activity of slightly acidic electrolyzed water (SAEW) depends on pH, available chlorine concentration (ACC), and organic demand [58,59]. Hypochlorous acid (HOCl) oxidizes susceptible microbial components. Electrolyzed water (EW) formulations are not chemically interchangeable: slightly acidic (SAEW), low-concentration acidic (LcEW), and alkaline forms differ in pH and chlorine speciation. In combined preservation systems, other interventions can contribute to storage outcomes: a study of fresh-cut apple, for example, paired SAEW with a chitosan–apple-essence coating [60]. Organic matter consumes available chlorine and may contribute to byproduct formation, affecting both treatment efficacy and chemical safety.

Organic matter released by cut tissues consumes active chlorine and can reduce washing efficacy across successive batches [59]. ACC, pH, contact time, and water reuse determine the exposure received by the food. Ultrasound can improve cleaning or transport, but chlorine replenishment remains a separate requirement [58]. Monitoring and dosing systems must account for changes in food loading and organic demand.

The DFU–EW mushroom study is a food-level example of this distinction: it used alkaline EW at pH 9.18 rather than SAEW [61]. Dual-frequency ultrasound (DFU) at 27 °C for 32 min and 67.5 W/L inhibited browning and maintained firmness over seven days. The study measured PPO properties as well as storage quality, so enzyme-related changes could be assessed alongside product performance.

### 3.4. High-Pressure Processing (HPP) and Pulsed Electric Fields (PEF) as Applicability Boundaries

HPP and PEF provide useful contrasts in matrix suitability. Pressure-transmitting purées and electrically conductive cut tissues differ from porous whole mushrooms in their exposure characteristics and susceptibility to structural damage. The following comparison focuses on these differences.

High-Pressure Processing

**Mechanism**. HPP can change protein and membrane organization, but the response of a specific enzyme depends on pressure, temperature, time, and matrix [62,63]. Molecular simulations or purified-protein changes support mechanistic interpretation, not a universal prediction of quality retention.

**Applicable matrices**. Studies of liquid and homogenized foods describe the nutritional and rheological outcomes of HPP [64,65]. Their applicability to fresh-market mushrooms is limited by differences in tissue structure and the importance of retaining an intact appearance.

**Limitations**. Tissue architecture and treatment conditions determine the textural response to HPP [66,67,68]. Pressure-induced exudation or loss of firmness can offset microbial or enzyme control in products valued for their intact structure. Efficacy and texture therefore need to be measured in the same food and pressure–temperature regime.

Pulsed Electric Fields

**Mechanism**. PEF alters membrane permeability through electrical exposure, with outcome determined by field strength, pulse characteristics, conductivity, and geometry [69]. Electroporation used to facilitate extraction or drying is not synonymous with preservation of an intact fresh product.

**Applications and limitations**. PEF-assisted extraction increases access to phytochemicals [70], while drying studies exploit changes in mass transfer [71,72]. These applications differ from fresh-product preservation, where increased permeability can affect texture and water retention during storage.

A study of fresh-cut apple examined PEF treatment in the Delicious cultivar after comparing the storage quality of five cultivars [73]. Thus, PEF can be useful in some solid-produce applications, although cut geometry and conductivity limit extrapolation to whole mushrooms.

**Figure 2:**
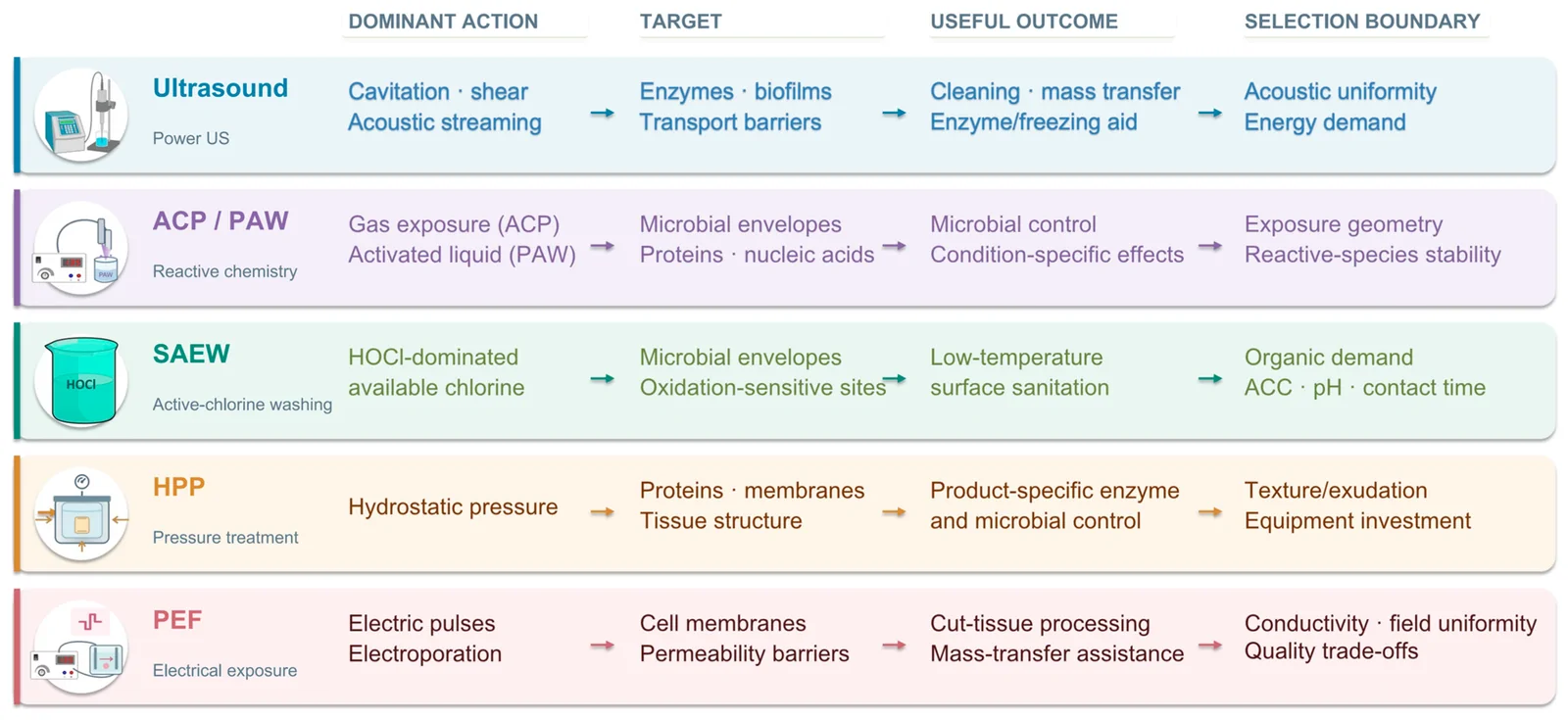
Mechanism-based comparison of non-thermal technology groups by dominant action, biological target, useful outcome and selection boundary. Gas-phase plasma exposure and activated-liquid treatment are distinguished within the ACP/PAW group. US, ultrasound; ACP, atmospheric cold plasma; PAW, plasma-activated water; SAEW, slightly acidic electrolyzed water; HPP, high-pressure processing; PEF, pulsed electric fields; HOCl, hypochlorous acid; ACC, available chlorine concentration.

Figure 2 organizes the five technology groups by dominant action, target, useful outcome and selection boundary. It distinguishes gas-phase ACP exposure from PAW-mediated liquid treatment and highlights acoustic uniformity, reactive-species stability, chlorine demand, tissue damage and electrical conductivity as different constraints. This mechanism-based overview complements the primary food studies in Table 1 and the process validation criteria in Table 2; it is not a quantitative ranking of efficacy or readiness.

## 4. Combination Treatments: Complementarity, Synergy Tests, and Process Windows

Combined treatments may address mass transfer, biofilm, or organic load limitations, but a larger effect than either single treatment does not by itself demonstrate synergy. The examples below are therefore described as combined or complementary unless the source study tested an additive expectation or treatment interaction.

The practical value of a hurdle treatment therefore depends on sequence, dose, matrix, comparator, microbial endpoint, quality trade-off, and energy demand, which should be reported together before an approach is considered transferable or scalable.

### 4.1. Physicochemical Complementarity in Sonication-Assisted Cold Plasma

Sonication-assisted cold plasma (SACP) treatments differ in physical configuration [74]. Airborne acoustic ultrasound with gas plasma and ultrasound with PAW in a liquid bath produce different exposure conditions. Ultrasound–electrolyzed water systems add a further distinction in oxidant chemistry. The contact medium determines the mechanical effects and active species available at the treatment target.

**Figure 3:**
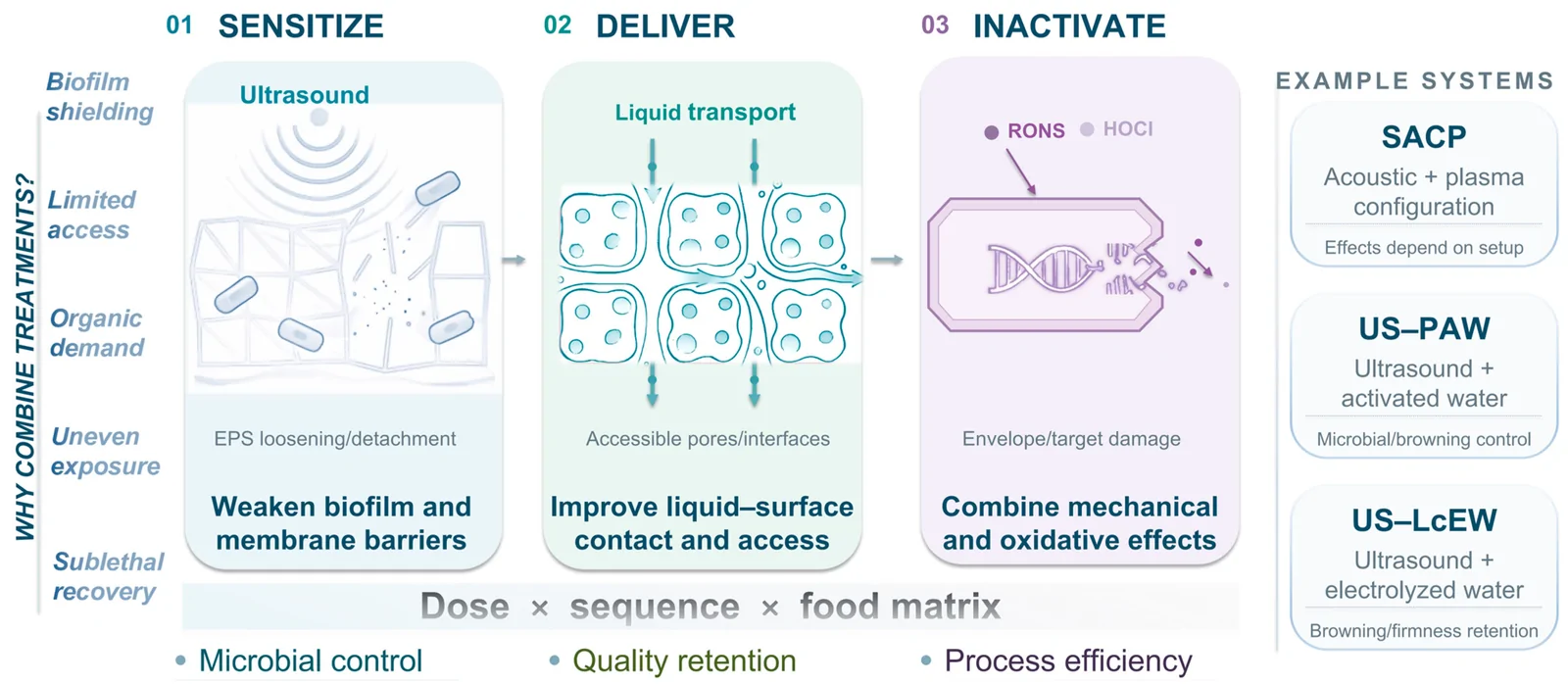
Conceptual framework for ultrasound-assisted combined treatments, organized as sensitization (biofilm loosening), improved liquid access, and microbial target damage. RONS and HOCl denote alternative, system-dependent oxidative chemistries, not obligatory co-occurring agents. Outcomes depend on dose, sequence and food matrix; the scheme does not establish universal synergy. EPS, extracellular polymeric substances; RONS, reactive oxygen and nitrogen species; SACP, sonication-assisted cold plasma; US–PAW, ultrasound with plasma-activated water; US–LcEW, ultrasound with low-concentration acidic electrolyzed water.

Figure 3 summarizes the rationale for combining treatments as sensitization, improved access and target damage. The diagram distinguishes biofilm loosening, liquid–surface contact and mechanical or oxidative effects, while showing that dose, sequence and food matrix govern the balance between microbial control, quality retention and process efficiency. SACP, US–PAW and US–LcEW are example configurations, not interchangeable chemistries or proof of universal synergy.

**Transport and exposure**. Ultrasound-enhanced mass transfer has been demonstrated in model separation systems [75]. During food washing, cleaning and liquid movement may similarly improve access to surface-associated microorganisms. However, transport into a particular pore or biofilm depends on surface geometry, liquid flow, and reactive-species lifetime; results from model systems provide mechanistic context rather than direct evidence of deep oxidant delivery in mushrooms.

**Configuration-specific evidence**. Atmospheric air plasma combined with airborne acoustic ultrasound has been studied against bacterial biofilms [76]. In kale, plasma-sonic treatment instead used PAW as the ultrasound medium [77]. Differences in exposure medium, target geometry, and measured endpoint limit direct comparison of these configurations.

### 4.2. Composite Washing Systems: US–PAW and US–SAEW

Ultrasound with active washing liquids offers a testable complementarity: mechanical action may improve access while the liquid chemistry supplies antimicrobial activity. A grape study of ultrasound with SAEW illustrates a berry-associated surface/wettability application, which must be distinguished from wounded lettuce or sliced mushrooms [78].

**US–PCPAW in fresh-cut lettuce**. Wang et al. tested iceberg lettuce inoculated with *Escherichia coli* O157:H7, non-O157 *Escherichia coli*, and *Salmonella* Typhimurium [79]. Treatment combined 28 kHz, 300 W ultrasound with pulsed-control PAW (PCPAW) for 1 min and assessed microbial reduction, transfer, browning, and sensory quality. The reported 27–33% cross-contamination value was a study-defined metric relative to the control, not an estimate of contamination incidence in commercial operations. Strawberry washing studies provide a separate surface treatment example [80], with different food structure and treatment conditions.

**Electrolyzed water with ultrasound in sliced mushrooms**. Wu et al. treated fresh-sliced button mushrooms with LcEW, with or without ultrasound, for 3 min before storage at 5 °C for 8 days [81]. The combined treatment delayed browning and maintained firmness; microbial loads and biochemical indicators were also measured. At the end of storage, total soluble protein and total phenolic content were 1.49 and 1.24 times the control values, respectively. Ultrasound-assisted SAEW freezing has been investigated separately in matsutake mushrooms [82]. Refrigerated washing and freezing have different process objectives and require separate evaluation of microbial control, tissue integrity, and quality.

### 4.3. Matrix-Specific Synthesis and Selection Rules

**Mushrooms**. Porous hyphal tissues and the importance of fresh appearance constrain treatment intensity. PAW studies on button mushrooms and LcEW–US treatment of slices measured storage outcomes, whereas ultrasound treatment of commercial PPO in buffer measured enzyme susceptibility [47,53,54,81]. Slicing changes treatment access and organic release, so doses effective on slices may not perform similarly on intact mushrooms. A practical treatment window needs to combine microbial reduction with acceptable firmness and browning in the intended product form.

**Berries**. Strawberry washing and blueberry cold plasma studies address different surface and quality responses [80,83]. In the blueberry experiment, six discharge/rest cycles were followed by eight days at 25 °C, with measurements of firmness and redox-associated gene expression; skin integrity, softening, and water loss determine whether an antimicrobial treatment remains acceptable. In grapes, US–SAEW reduced natural bacterial and mold/yeast counts by 2.23 and 2.76 log CFU/g, respectively, and improved storage quality indicators [78]. These surface wettability effects are most relevant to intact skin rather than wounded mushroom tissue.

**Fresh-cut produce**. Exposed tissue improves access to active washing liquids but also increases leakage and chemical demand. Lettuce US–PCPAW and apple SAEW–coating studies illustrate different responses to this geometry [60,79]: the former examined a combined wash and microbial transfer, whereas the latter included a coating barrier during storage. The apple study assessed PEF only in the selected Delicious cultivar, so its findings require validation in other cultivars [73], so comparisons are most informative when microbial and quality endpoints are measured under matched processing conditions.

Table 1 illustrates three considerations for treatment selection: correspondence between the tested and intended food, the balance between microbial and quality outcomes, and validation at the required processing scale. The available studies cover these aspects unevenly, so laboratory efficacy alone is insufficient to select an industrial treatment where resource use or process uniformity remains unmeasured.

## 5. What Omics Can—and Cannot—Resolve

Transcriptomic and metabolomic studies identify treatment-associated responses and candidate biomarkers, but their interpretation depends on species, tissue, exposure, and storage conditions. Plant and fungal findings are therefore considered separately, since similar quality outcomes can arise through different pathways.

### 5.1. Membrane Lipid Remodeling and Metabolic Responses

Liquid chromatography–mass spectrometry can characterize treatment-associated metabolites and lipids, but analytical coverage, identification confidence, and quantification vary by workflow [84,85]. Such measurements identify candidate responses or process markers rather than the pathway that causes quality retention.

**Wounding and lipid-associated responses**. Studies of fresh-cut potato, apple, and lettuce associate browning or storage conditions with changes in metabolic profiles [16,86,87,88,89,90]. Work on cucumber has examined phospholipase D and lipoxygenase responses to mechanical injury [91]. Together, these findings implicate membrane-associated metabolism in deterioration, but the relevant metabolites and timing vary among commodities. Their contribution to mushroom browning requires fungal tissue-specific evidence.

**Release, transformation, and synthesis of phenolics**. Metabolic markers of processing have been identified in strawberry and apple purées [92]. Studies of wine made from sonicated grapes and of phenolic regulation in *Salvia miltiorrhiza* provide further evidence that phenolic profiles depend on processing and biological context [93,94]. Strawberry PEF/plasma experiments and work on blended juice extend these comparisons to other matrices [95,96]. Changes in total phenolic content may reflect release, transformation, or altered synthesis; distinguishing these routes requires compound-specific and functional measurements.

**Energy metabolism**. Elevated-CO_2_ studies in mushrooms and plasma or chemical treatment studies in plant commodities link energy status with senescence [97,98,99]. Ultrasound-treated apples and research on elicitation in plant foods provide additional contexts for examining metabolic responses [100,101]. ATP-associated and membrane potential changes can help characterize treatment responses, although their causal contribution to preservation depends on the commodity and intervention.

A possible acclimation window must therefore be established for each commodity and treatment rather than assumed from a generic dose–response model.

### 5.2. Transcriptomic Associations and Candidate Signaling Networks

Mushrooms and plant commodities remain metabolically active after harvest, but their responses differ biologically. Transcriptomic and metabolomic studies of rambutan, blueberries, and PAW-treated plant tissues identify treatment-associated genes and processes [83,102,103]. These measurements describe responses in the tested plant matrices; establishing a signaling cascade also requires evidence at the protein and functional levels.

RNA-Seq combined with physiological measurements has been used to investigate ultrasound-associated softening responses in strawberries [104]. Linking expression profiles to the measured phenotype provides candidate mechanisms under the tested dose and storage conditions.

**Antioxidant-associated responses**. Studies in litchi, strawberry, and blueberry matrices report treatment-associated ROS metabolism or gene expression responses [105,106,107]. These observations motivate joint measurements of microbial control and host oxidative damage. Expression of SOD, CAT, APX, or GR is not, by itself, proof of improved enzyme activity, net antioxidant capacity, or causal quality protection.

**Phenylpropanoid-associated responses**. PAL, C4H, and 4CL belong to pathways studied in plant commodities and plant defense systems [108,109,110,111]. These pathways may explain selected plant observations, but their occurrence, regulation, and relevance cannot be transferred to edible fungi solely because both groups show browning or antioxidant responses.

Candidate signaling networks. Upstream Ca^2+^, MAPK, and transcription factor relationships are hypotheses that must be distinguished from measured transcription, phosphorylation, promoter binding, and treatment-dependent quality.

**Figure 4:**
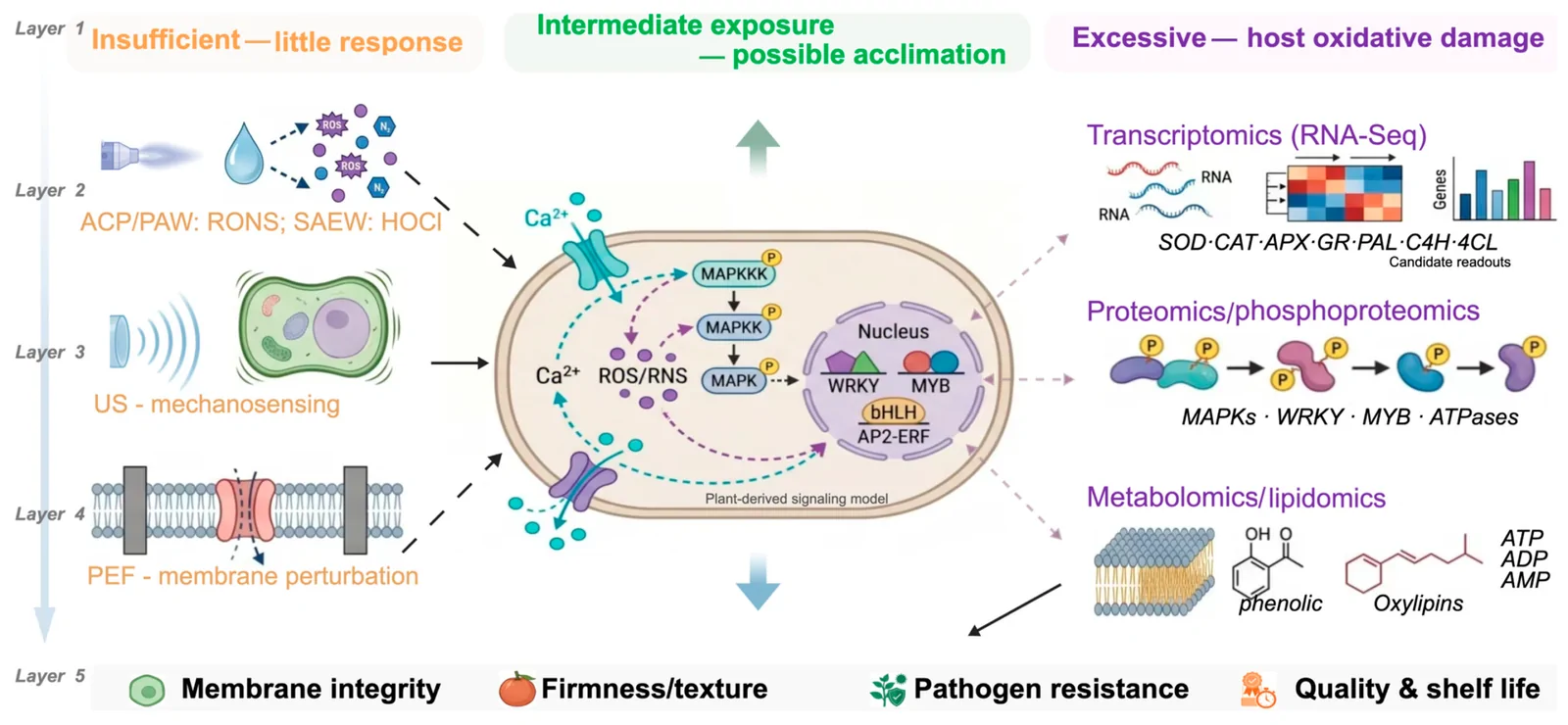
Conceptual integration of exposure intensity, treatment inputs, a plant-derived signaling model, and candidate omics readouts (transcriptomics, proteomics/phosphoproteomics, and metabolomics/lipidomics) with preservation-related outcomes. Insufficient exposure produces little response, intermediate exposure may elicit acclimation, and excessive exposure may damage the host. The central Ca^2+^–MAPK–transcription factor model is plant-derived, not a demonstrated conserved fungal pathway; candidate readouts do not establish causality. MAPK, mitogen-activated protein kinase; RNA-Seq, RNA sequencing; US, ultrasound; PEF, pulsed electric fields; RONS, reactive oxygen and nitrogen species; HOCl, hypochlorous acid.

Studies of plant stress, phytohormone elicitation, and transcription factor regulation provide possible links between treatment perception and defense responses [112,113,114,115]. The full sequence from non-thermal exposure through Ca^2+^ influx, MAPK activation, and transcription factor binding to quality retention remains unestablished across the commodities considered here. Transcript abundance, promoter binding, and phosphorylation describe different levels of regulation; causal inference requires functional perturbation linked to the relevant quality endpoint. Figure 4 combines exposure intensity, a plant-derived signaling model, and candidate omics measurements. This model can guide hypothesis testing, but its transfer to mushrooms requires direct fungal evidence.

Current omics evidence is therefore most useful for generating candidate biomarkers and defining commodity-specific dose–quality windows; a shared Ca^2+^–MAPK–transcription factor cascade has not been demonstrated for the treatments reviewed here.

## 6. Safety, Scale-Up, Sustainability, Regulation, and Acceptance

Promising laboratory effects do not constitute industrial readiness. Translation requires validation of microbial safety, quality retention, treatment uniformity, throughput, resource demand, equipment cost, regulatory classification, and consumer acceptance for the intended commodity and process.

### 6.1. Viable but Non-Culturable (VBNC) Cells and Validation Strategies

VBNC formation depends on the organism and treatment. *Staphylococcus aureus* lost culturability and subsequently resuscitated after prolonged low-dose SAEW exposure in one study [116]. Broader reviews describe stress- and organism-dependent behavior [117,118,119]. These observations support viability-informed process validation, while the relevance to short produce-washing regimes depends on the food matrix, exposure, and recovery conditions.

Culture negativity alone does not prove cell death, while membrane integrity or a molecular signal alone does not prove infectivity or resuscitation potential [120,121]. Culture-based enumeration should therefore be complemented by appropriate viability-oriented methods and, where relevant, recovery experiments. PMA-qPCR can reduce contributions from membrane-compromised cells, flow cytometry can characterize staining-defined populations, and RNA assays can assess selected transcripts; none independently identifies every VBNC cell or establishes pathogenicity [122,123,124]. A robust validation design separates viable-cell indicators, culturability, and recovery under defined conditions.

### 6.2. Life-Cycle, Cost, and Scale-Up Considerations

Life-cycle and cost comparisons should use a common functional unit, such as processing one kilogram of product to a specified microbial reduction while retaining predefined color or firmness. Electricity, water, gases or chemicals, cleaning, throughput, equipment lifetime, and product yield should be included; current heterogeneous studies do not support a universal ranking.

Scale-up performance depends on the delivered treatment and usable product yield. Ultrasound energy demand varies with geometry, attenuation, temperature control, and throughput [125,126]. HPP requires pressure-rated equipment and substantial investment; its cost per kilogram also depends on loading and cycle utilization [127]. Plasma uniformity depends on electrode configuration, product loading, and exposure geometry [128]. Table 2 compares these resource and validation requirements alongside microbial and quality endpoints.

Reported energy and cost figures indicate the scale of these requirements, although their functional units and system boundaries differ. High-pressure processing is commonly estimated at 0.1–0.5 kWh per kilogram of product, depending on pressure, holding time, and whether the boundary includes the intensifier and cooling system [129]. A prototype electrolyzed-water generator has been evaluated for energy consumption and production cost [130]. Plasma-activated water production costs have been estimated at roughly 0.01–0.27 € per liter of activated water in laboratory reactors, which is several times more than chemically prepared solutions of similar composition [131]. For ultrasound washing, reported values are usually expressed per liter of wash water or as percentage savings rather than per kilogram of produce, and product yield and cleaning are rarely quantified together, so cross-technology comparison requires an explicit water-to-product ratio and a stated system boundary.

Engineering priorities include continuous or thin-layer reactors, validated treatment uniformity, online dose monitoring, and matrix-specific scale-up. These needs apply differently to washing, plasma, pressure, and electric-field systems and should not be reduced to one preferred equipment route.

### 6.3. Packaging and Cold-Chain Integration

Packaging and cold-chain controls define the post-treatment system boundary by limiting recontamination, moisture loss, and temperature abuse. Proposed combinations with active coatings or digital indicators require validation as integrated systems rather than inference from separately demonstrated modules.

Active coatings, antimicrobial packaging, and modified atmospheres can complement post-treatment storage [132,133,134]. Sensors and indicators monitor selected environmental conditions or spoilage-related changes [135,136,137]; their signals must be interpreted alongside microbial measurements, because a freshness response does not necessarily indicate pathogen control.

### 6.4. Regulation and Consumer Acceptance

Regulatory classification and consumer acceptance depend on jurisdiction, intended use, treated product, process chemistry, and labeling, and should be assessed for the specific technology–commodity combination rather than generalized across non-thermal treatments.

#### 6.4.1. Jurisdiction- and Use-Specific Regulatory Assessment

In the United States, FDA Food Contact Notification 1811 applies to IET, Inc., DBA EcoloxTech, and its customers under specified conditions. Available free chlorine must not exceed 60 ppm. For the listed fruits, vegetables, and other foods intended to be consumed raw, treatment is followed by either a 10 min drain step or a potable-water rinse [138]. These conditions apply to the notified use rather than all SAEW systems. In the European Union, novel food status depends on the food and process. A process not used before 15 May 1997 may fall within the framework if it causes relevant significant compositional or structural changes. Operators must verify status and seek consultation when needed; foods classified as novel require authorization and Union-list inclusion before marketing [139,140]. The EFSA provides scientific assessment, whereas the European Commission makes authorization decisions. Classification of ACP- or PAW-treated foods therefore requires product- and process-specific assessment.

#### 6.4.2. Consumer Perceptions of Non-Thermal Technologies

Consumer responses to non-thermal processing depend on terminology, familiarity, perceived benefit, and the information provided [141,142]. Communication should distinguish the actual treatment chemistry and product use from unrelated technologies, without presuming that every consumer holds the same misconceptions. Transparent descriptions of measured benefits, uncertainties, residues, and applicable rules are more informative than generic claims that a process is inherently green or safe.

## 7. Conclusions and Future Directions

Treatment selection depends on product structure and the intended preservation endpoint: LcEW–US treatment of sliced mushrooms and US–PCPAW washing of fresh-cut lettuce reduced microbial load while maintaining measured quality attributes, whereas the optimum dose for intact mushrooms and whole berries remains unresolved. Microbial control and quality retention must be assessed within the same process window, because greater microbial reduction can be offset by damage to texture, appearance, or yield. Combined treatments require comparison with an additive expectation, and cost, energy use, and scale-up feasibility require a common functional unit. Enzyme buffer and juice results do not replace experiments on the intended food, and priority validation should include matched microbial–quality dose windows, treatment uniformity at realistic loading, repeated-batch water chemistry, and product yield. Regulatory classification and consumer communication still need to be resolved for the specific product and jurisdiction.

Omics studies identify candidate responses whose contribution to preservation requires functional testing. Plant antioxidant, phenylpropanoid, and signaling findings offer hypotheses rather than established fungal pathways; linking defined exposures with quality phenotypes and targeted functional measurements would help resolve these mechanisms.

The matrix–target–readiness framework links food-matched efficacy, quality trade-offs, and process validation. Its value lies in identifying which options are best supported for a particular product and which evidence is still required before scale-up, rather than selecting one technology for all commodities.

**Table 1:** Representative non-thermal preservation studies in mushrooms, berries, and fresh-cut produce. Each row gives the treatment and storage conditions, the measured outcome, and the limits of the study. Differences in product form, treatment, and storage conditions preclude direct ranking of efficacy across studies.

Technology	Food/Product Form	Treatment/Storage Conditions	Measured Result	Scope and Limitations	Reference
DFU–EW	Fresh-cut *A. bisporus*	27 °C, 32 min; alternating 30:30 s dual-frequency operation; 67.5 W/L; EW at pH 9.18; storage evaluated for 7 days.	Browning inhibited and firmness maintained; mean relative PPO activity approximately 60% during storage.	Alkaline EW (pH 9.18), distinct from SAEW; laboratory study with a seven-day storage evaluation.	[61]
PAW	Button mushrooms	Plasma activation: 5–25 min.	Surface *E. coli* reduction ranged from 0.11 to 1.32 log10 CFU/g across activation conditions; the 20 min activation group performed best for several quality indicators.	Effects depended on water-activation conditions; activation duration alone does not specify food-contact exposure.	[53]
PAW	Button mushrooms	PAW soaking followed by storage for 7 days at 20 °C.	Reported bacterial and fungal count reductions during storage were 1.5 and 0.5 log, respectively; softening was delayed, without significant change in color, pH or antioxidant properties.	Ambient storage at 20 °C; applicability to refrigerated distribution remains to be evaluated.	[54]
LcEW + US	Fresh-sliced button mushrooms	3 min pretreatment; 5 °C storage for 8 days; compared with tap-water control and LcEW alone.	Browning delayed and firmness maintained; microbial loads and biochemical indicators assessed. End-storage soluble protein and total phenolic content were 1.49 and 1.24 times control, respectively.	Laboratory study of sliced mushrooms during refrigerated storage; transfer to whole mushrooms or freezing processes requires separate evaluation.	[81]
US + SAEW	Whole grapes	40 kHz, 50 W/L; 30 s US plus 270 s agitation; 25 °C wash; 1:6 solid/liquid; storage at 4 ± 1 °C.	Reported bacterial and mold/yeast reductions were 2.23 and 2.76 log CFU/g; lower weight loss and better firmness/quality retention than the water-cleaned comparison were reported.	Whole-grape skin and wettability effects; natural bacterial and mold/yeast counts rather than inoculated-pathogen challenge endpoints.	[78]
US–PCPAW	Fresh-cut iceberg lettuce	28 kHz, 300 W; 1 min; 1:10 solid/liquid; 120 rpm agitation; pulsed-control PAW distinguished from conventional PAW.	Pathogen reductions of 2.9–3.1 log CFU/g for *E. coli* O157:H7, non-O157 *E. coli*, and *S.* Typhimurium; browning and sensory endpoints also assessed.	Laboratory lettuce-washing study; cross-contamination measured relative to the experimental control.	[79]
ACP (DBD)	Blueberries, cv. Emerald	CP-6: six 10 s discharge/10 s rest cycles; storage at 25 ± 0.5 °C and 85% RH for 8 days.	Hardness was better maintained; H_2_O_2_ was reported 12.1% lower; transcriptome changes involved antioxidant and cell wall-associated genes.	Single-cultivar plant study; expression measurements identify associated responses rather than a causal fungal signaling pathway.	[83]
PEF	Fresh-cut Delicious apple	1, 3 or 5 kV/cm; 200 μs, 500 Hz; reported 1 h treatment at room temperature; storage at 4 °C for 10 days.	At 3 kV/cm, day-10 PPO and POD activities were 44.61% and 36.48% lower than control, respectively; higher field strengths addressed different endpoints.	Laboratory, variety-specific study with a 1 h treatment period; process time and applicability to other products require further evaluation.	[73]

Abbreviations: US, ultrasound; DFU, dual-frequency ultrasound; EW, electrolyzed water; SAEW, slightly acidic electrolyzed water; LcEW, low-concentration acidic electrolyzed water; PAW, plasma-activated water; PCPAW, pulsed-control PAW; ACP, atmospheric cold plasma; DBD, dielectric barrier discharge; PEF, pulsed electric fields; PPO/POD, polyphenol oxidase/peroxidase. All Table 1 examples are laboratory studies; their results do not by themselves establish pilot or industrial performance.

**Table 2:** Process considerations for matrix-dependent selection of non-thermal treatments. Row labels correspond to the technology groups discussed in Section 3 and Section 4. The comparison covers efficacy, quality, resource demand, and scale-up requirements; it is qualitative, and published energy and cost figures for selected technologies are compiled in Section 6.2.

Treatment	Efficacy and Quality Endpoints	Resource Demand and Cost Factors	Scale-Up Constraints
US washing	Food surface microbial reduction, firmness, color, and tissue leakage; limited transferability of enzyme buffer results.	Ultrasound electricity, cooling, bath volume, and cleaning per kilogram of usable product.	Acoustic exposure at realistic loading; geometry, batch reproducibility, and quality at the least- and most-exposed positions.
Direct ACP	Discharge/contact geometry, food surface, microbial reduction, and, where relevant, residue degradation and residual toxicity.	Discharge electricity, gas supply, cooling/ventilation, treatment duration, auxiliary demand, and product yield.	Exposure uniformity, loading, surface shadowing, throughput, residues, and sensory limits.
PAW/US–PAW	Activation chemistry at food contact, microbial transfer, and food quality; separate activation and washing times.	Water activation, ultrasound where used, water replacement, chemistry decay, cooling, and cleaning.	Organic demand, active-species decay, water reuse, repeated batches, and treatment performance at production flow rates.
SAEW/LcEW with or without US	pH, ACC, organic load, and contact time in relation to decontamination, browning, and firmness.	Water, salt/acid, electrolysis, replenishment, rinsing, ultrasound where used, and usable product yield.	ACC/pH control, chemical consumption, byproducts, bath reuse, loading, and recontamination.
HPP: intact fresh products	Microbial/enzyme control, firmness, and exudation in the same product; tissue architecture limits transfer from juices and purées.	Pressure equipment, cycle/loading utilization, water, cooling, and packaging.	Retention of acceptable tissue structure after pressure cycling under realistic loading.
PEF: cut tissues and liquids	Variety, conductivity, microbial and quality responses; distinction between extraction/drying pretreatment and storage preservation.	Delivered electrical energy, pulse generation, cooling, electrodes, pretreatment, and yield or texture losses.	Field uniformity, conductivity variation, heating, electrode effects, and throughput in the intended food geometry.

## Data Availability

No new data were created or analyzed in this study. Data sharing is not applicable to this article.
